# DOCEST—fast and accurate estimator of human NGS sequencing depth and error rate

**DOI:** 10.1093/bioadv/vbad084

**Published:** 2023-07-18

**Authors:** Lauris Kaplinski, Märt Möls, Tarmo Puurand, Maido Remm

**Affiliations:** Institute of Molecular and Cell Biology, University of Tartu, Riia 23, Tartu 51010, Estonia; Institute of Molecular and Cell Biology, University of Tartu, Riia 23, Tartu 51010, Estonia; Institute of Molecular and Cell Biology, University of Tartu, Riia 23, Tartu 51010, Estonia; Institute of Molecular and Cell Biology, University of Tartu, Riia 23, Tartu 51010, Estonia

## Abstract

**Motivation:**

Accurate estimation of next-generation sequencing depth of coverage is needed for detecting the copy number of repeated elements in the human genome. The common methods for estimating sequencing depth are based on counting the number of reads mapped to the genome or subgenomic regions. Such methods are sensitive to the mapping quality. The presence of contamination or the large deviance of an individual genome from the reference may introduce bias in depth estimation.

**Results:**

Here, we present an algorithm and implementation for estimating both the sequencing depth and error rate from unmapped reads using a uniquely filtered *k*-mer set. On simulated reads with 20× coverage, the margin of error was less than 0.01%. At 0.01× coverage and the presence of 10-fold contamination, the precision was within 2% for depth and within 10% for error rate.

**Availability and implementation:**

DOCEST program and database can be downloaded from https://bioinfo.ut.ee/docest/.

**Supplementary information:**

Supplementary data are available at *Bioinformatics Advances* online.

## 1 Introduction

Sequencing depth (of coverage) can be defined as the average number of times each nucleotide of source DNA is present in the output file (Sims *et al.*, 2014). For full-genome sequencing, it usually ranges from 30× for the analysis of individual DNA from blood or saliva for mutation discovery to 0.001× for analyzing ancient DNA.

The need for precise depth of coverage estimation arises in the analysis of genomic variance, especially if relatively short next-generation sequencing (NGS) reads are used. For example, estimating the copy number of indels requires comparing the sequencing depth of the relevant region with surrounding regions (Janevski *et al.*, 2012). However, because of stochastic variance in coverage, the ratios of sequencing depths often deviate from clear integer ratios, although copy numbers should be integers. Especially for larger copy numbers a maximum likelihood, or similar, model is used to choose the best estimation. The prediction of such a model depends on the estimation of the depth of coverage of the surrounding regions. Recently there has been a lot of interest in describing the long repeated regions (centromeres, telomeres and heterochromatin) of the genome, where the copy numbers are in thousands or more.

A similar problem arises for SNV detection, where the model is used to predict the likelihood that a given set of diverging reads is the result of an SNV, instead of mismapping or sequencing errors.

Although conceptually trivial, the precise determination of sequencing depth has turned out to be tricky (Liu *et al.*, 2013; Roca *et al.*, 2019). One cannot simply count the total number of nucleotides in generated FASTQ files because of the possibility of sample contamination. Counting the reads that are mappable to the human reference genome works better, but still deviates from the correct result if the given individual has large (in several megabases) insertions or deletions, resulting in under or overestimation of the depth. If the individual has genomic regions that are not present in the reference, the corresponding reads are not mapped. In addition, if the sample is contaminated with the DNA of other organisms, the mapper may align some contaminant reads to the human genome, thus overestimating the depth of coverage. This is especially relevant in ancient DNA analysis where as little as 1% may come from the target individual, the majority being soil microbes and fungi.

Another problem is that the precise genome length of an individual is generally not known, as the lengths of genomes of different humans may vary up to 6% (Sun *et al.*, 2018). Thus, even if the number of mapped nucleotides is correct, the number of total nucleotides is not the same as in the reference genome and the estimation is wrong.

These problems can be attributed to the fact that the human reference genome is just one random individual. Mapping reads to the reference highlights the common (or similar) parts, but leaves the novel parts and large differences unmapped.

We present a method and program DOCEST (Depth Of Coverage ESTimator) that can determine the precise sequencing depth of the human genome and the error rate from unmapped reads by comparing *k*-mers to the universally conserved part of the genome at various depths and contamination levels. In a simulation, the estimation error was less than 2% even at depth 0.001 and less than 10% at the 10-fold contamination by eukaryotic and prokaryotic DNA.

## 2 Methods

### 2.1 The general idea

The idea of DOCEST is counting unique and universal tag *k*-mers from the sample. We define a unique and universal *k*-mer as a *k*-mer that is present exactly once in the genomes of most humans. The ratio of the total count of tag *k*-mers count in the sample to the number of all tag *k*-mers corrected with the read length and the number of sequencing errors is the depth of coverage of the sequencing sample. By counting only unique and universal *k*-mers, the method should be robust against both the large-scale copy number variations and the differences in the length of genome, as these commonly involve changes in repeated regions.

To estimate errors, unique random mismatched variants of the tag *k*-mers (error *k*-mers) are counted. By uniqueness we mean that a given mismatched *k*-mer is neither present in the reference genome nor can appear by a single substitution of any other *k*-mer than the original one. The ratio of error *k*-mers to the number of tag *k*-mers corrected with the read length gives us the proportion of sequencing errors.

The precise formulas used are given below.

### 2.2 *K*-mer selection

To estimate sequencing depth, we used the *k*-mers from NIPTMer package (Sauk *et al.*, 2018) with three additional filtering steps:

First, we removed all *k*-mers that were not present or not unique in the CHM13 genome.

Then we removed all *k*-mers that had hamming distance below 2 with the closest other *k*-mer in the human GRCh38p10 reference genome.

Then we split the reference genome into 100 bp windows and kept only one *k*-mer in every third window, thus ensuring that the distance between any two *k*-mers was at least 200 bp. This helps to reduce stochastic noise at low sequencing depths by limiting the maximum contribution of one NGS read to one tag *k*-mer (2 for paired end reads).

The final list contained 6885172 tag *k*-mers. *K*-mers were annotated with the information of chromosome and 1 Mbp window inside chromosome to allow sub-chromosomal depth estimation in the future.

To estimate the number of sequencing errors we created another set of *k*-mers, by inserting random errors into the first list. For each *k*-mer from the set of tag *k*-mers, four variants with one mismatch were created.

Increasing the number of error *k*-mers increases the error rate estimation precision, but results in larger list files. In our opinion, the relative precision of error estimation can safely be an order of magnitude lower than that of depth estimation. The former is mostly needed to estimate the number of *k*-mers ‘lost’ because of errors and the number of erroneous *k*-mers that can appear in sequencing data. As the error rate of most state of the art NGS technologies is less than 1%, even 1% error in estimation of error rate results less than 0.01% error in the estimation of coverage. In addition, the problem of erroneous *k*-mers, in our experience, cannot be easily modeled by the global error rate, as most erroneous *k*-mers that influence various analyses come from repeated regions of the genome, sometimes because of more than one sequencing error.

Thus, we decided to use four variants per *k*-mer, which resulted in enough error *k*-mers found to estimate error rate with 5% precision even for coverage as low as 0.001.

Both the placement of mismatch and substitution nucleotide were chosen randomly. Then only those *k*-mers having hamming distance greater than 2 with the closest genomic *k*-mers (other than the original one) were kept.

The final list contained 23597871 error *k*-mers.

### 2.3 Coverage and error rate computation

Both *k*-mer lists were concatenated into gmer_counter database. Gmer_counter (Pajuste *et al.*, 2017) counts *k*-mers in FASTQ files from a predefined set, arranged into clusters (nodes).

There are always fewer *k*-mers than nucleotides in sequence because only continuous sequences of k nucleotides are counted as a single *k*-mer by sliding window. At the end of reads and near overlapping ‘N’ there is a discontinuity in sliding window movement and thus less *k*-mers are generated.

The sequencing depth and error rate were estimated with the following formulas:


$$P_{e}=\frac{D_{\mathrm{err}}}{\left(D_{\mathrm{err}}+\frac{D_{\mathrm{tag}}}{3}\right)}.$$

*P*_e—_ the rate of sequencing errors (false nucleotide, not N). Here, we expect sequencing errors being unbiased—i.e. each nucleotide in genome has an equal probability (*P*_e_) to give erroneous value in each read it appears in.

*D*_tag_—tag *k*-mer depth—i.e. the number of tag *k*-mers seen divided by the total number of tag *k*-mers in list.

*D*_err_—error *k*-mer depth—i.e. the number of error *k*-mers seen divided by the total number of error *k*-mers in list.

The logic behind the formula is the following: With probability *P*_e_ there is an error in sequencing one nucleotide, resulting in one of the three alternative nucleotides. This results in an erroneous *k*-mer that is not counted in tag *k*-mer set (because the edit distance between any two tag *k*-mers is greater than one). Most of those wrong *k*-mers are silently missed, but some are ‘captured’ by the list of error *k*-mers. The more detailed explanation of the derivation of error formula is given in Supplementary File S1.


$$D=\frac{D_{\mathrm{tag}}N_{\mathrm{nucl}}}{{\left(1-P_{e}\right)}^{k}N_{k\mathrm{mer}}}$$

where *D* is the depth of coverage; *k* is the *k*-mer length (25 in our analysis); *N*_nucl_ is the number of nucleotides in sample and *N*_kmer_ is the number of *k*-mers in sample.

To reduce the effect of a large contamination to the estimation if the contaminant contains the same or similar *k*-mers, we eliminated *k*-mers both from sample and from list if their count in sample exceeded cutoff. The cutoff was set so that the probability of seeing even one *k*-mer with such or higher count in the whole sample was less than 1%.

### 2.4 Simulation

Unfortunately, the true sequencing depth of actual samples is unknown due to possible contamination and genome size variation. Therefore, to evaluate the precision of the algorithm at low sequencing depths and the presence of contamination, we generated simulated reads.

We used the following assembled genomes:

1. T2T CHM13v2.0 telomere-to-telomere assembly of the CHM13 cell line (RefSeq: GCF_009914755.1).
2. *Penicillium chrysogenum* strain P2niaD18 genome (GenBank: CM002798.1).
3. *Mus musculus* assembly GRCm39 (RefSeq; GCF_000001635.27).
4. *Escherichia coli* strain K-12 (RefSeq; GCF_000800765.1).

NGS reads were simulated with a custom Perl program separately for each reference genome by choosing random 100 bp regions uniformly over the whole sequence. All N-s in reference were replaced with random nucleotides to avoid bias. Then, to simulate sequencing errors, each nucleotide in each simulated read was replaced with N with 1% probability or random another nucleotide with 1% probability. The simulation script is included in the downloadable package.

The simulated sequencing depths of the human genome varied from 0.001 to 0.333 (0.001, 0.0033, 0.01, 0.033, 0.1, 0.333). For each sample, we simulated the contamination by adding an equal (by the number of nucleotides) mixture of simulated reads of *P.chrysogenum*, *M.musculus* and *E.coli* so that the fraction of human reads in final sample varied from 0.1 (10× contamination) to 1 (pure human genome).

## 3 Results

### 3.1 Precision

**Figure 1.**
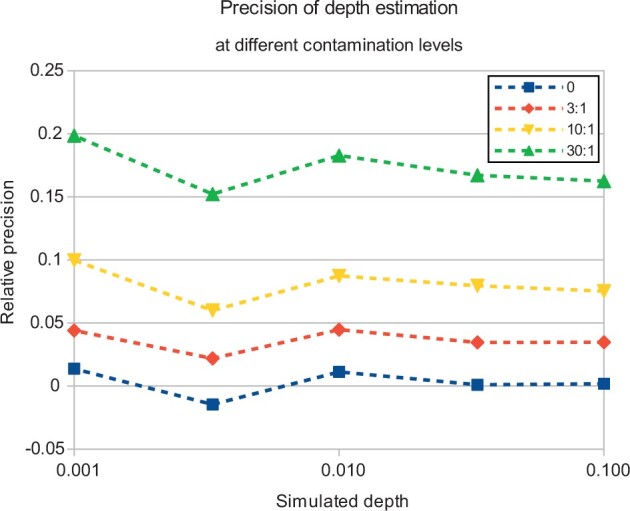
The precision of depth estimation at different depths and contamination levels. *X*-axis, the simulated depth of the sample. *Y*-axis, the relative difference between estimated and simulated depth. Different colored lines show the contamination level (the amount of contaminant DNA relative to the human DNA in base pairs)

First, we ran the program on the simulated low-depth dataset to get an estimation of the precision of the method. As was to be expected, the precision of depth estimation decreased with lower depth and larger contamination. The former can be attributed to stochastic noise. The latter is probably caused by identical or similar *k*-mers in the contaminant. Overall, the relative error of depth estimation varied from 0.1% at depth 0.333 and no contamination to 15% at depth 0.1 and 10× contamination. The comparison of results at different simulated depths and contamination levels is given in Figure 1.

The estimation of error rate is less precise than the estimation of depth. Here, the relative error ranged from 0.2% to 22%.

**Figure 2.**
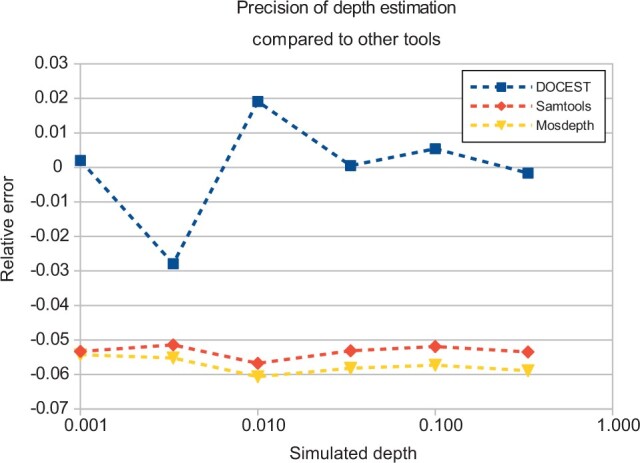
The precision of depth estimation of DOCEST compared with samtools and mosdepth. *X*-axis, the simulated depth of the sample. *Y*-axis, the relative difference between estimated and simulated depth

We also compared the DOCEST with two state-of-the-art depth estimation tools: Samtools (Li *et al.*, 2009) and Mosdepth (Pedersen and Quinlan, 2018). As both of these programs analyze already mapped reads in sam or cram format, reads were mapped on the human reference genome GRCh38.p140 (GenBank: GCA_000001405.29). The weighted average over all mapped regions were used as the estimation of total coverage. While DOCEST was the most precise at zero contamination, at higher contamination levels mapping-based tools outperformed it. Interestingly both Samtools and Mosdepth underestimated the true depth by about 4%. The results are shown in Figure 2.

The full results of simulated data are given in Supplementary Table S1.

In addition to simulation, we also analyzed two sets of real sequencing data: 10 low-coverage samples from medieval Europeans (Gretzinger *et al.*, 2022) and 10 high-coverage samples from the 1000 Genomes project (Auton *et al.*, 2015).

Again, the depth estimation from DOCEST was found to be 3–17% higher compared with mapping-based tools. The full results are given in Supplementary Table S2.

### 3.2 Performance

Most of the time of DOCEST is spent counting tag *k*-mers from sequencing reads. Thus, the total running time is dependent on the size of the source (FASTQ) files. On a workstation class computer with 32 Intel Xeon 2.4 GHz cores, processing the data with 30× coverage took 2 h, 0.33× coverage approximately 3 min.

Due to the different approach, the performance of DOCEST is not directly comparable to mapping-based tools. In raw comparison, DOCEST was 3–10 times slower than Samtools and Mosdepth. However, these tools rely on already mapped and indexed bam or cram files, the creation of which takes considerable time. On the other hand, mapping reads is a routine part of any NGS analysis, and bam or cram is the standard storage formats of NGS data. The full running times on simulated data are available in Supplementary Table S1.

## 4 Discussion

Knowing the true sequencing depth of the human genome allows one to estimate the length or copy number of variable regions. This is especially challenging for low-coverage sequencing, e.g. for ancient DNA or cell-free DNA analysis, and in the presence of contamination.

We demonstrate that by using a pre-filtered *k*-mer list, we can estimate the simulated sequencing depth reliably even at depth as low as 0.001, i.e. only 3 Mbp genomic reads, and the presence of eukaryotic and prokaryotic contaminants up to 10× the amount of human DNA. The error rate estimation is slightly less precise than the depth estimation. However, in this case, the importance of knowing the true error rate might not be as critical as understanding the sequencing depth. Therefore, a 10× contamination level is considered acceptable. By using only the conserved part of the genome for *k*-mer counting, DOCEST estimation should be robust against individual variations in genome size as long as the variation occurs mostly in repeated regions.

On simulated reads, we detected small but consistent differences with the estimation of DOCEST compared with mapping-based tools. This was probably caused by the differences between the simulated individual (CHM13V2.0) and the reference genome (GRCh38.p140). As CHM13V2.0 is a single haplotype of a real individual genome without gaps, it may contain certain regions not present in the reference genome, as indicated by a small number of unmapped reads. We expect that the genomes of real individuals also contain unique fragments and thus the mapping-based depth estimation may be lower than the real sequencing depth. In our opinion, this is an often overlooked aspect and needs to be kept in mind when working with whole genome sequences.

One potential drawback of our method is that our tag *k*-mer list was filtered only with the European population. To increase the reliability, additional filtering should be performed, removing all *k*-mers that are not unique and universal in other populations.

Although contamination with the DNA of another individual is also a potential problem for low-depth sequencing, DOCEST cannot filter it. As any two individuals differ only at a certain number of polymorphic positions, we do not see a way to separate their reads in low-coverage samples other than domain-specific approaches, such as using the degradation pattern of ancient DNA.

It is also well known that the sequencing depth is not uniform over the whole genome, depending on the relative GC content of each region. Although we tagged *k*-mers with 1 Mbp chromosomal window, the current tool does not allow the fine-grained depth estimation. In any case, we can expect the relative errors to be proportional to the number of tag *k*-mers in the region and thus the sub-genomic depths, if calculated, to be much less precise.

Our method is currently restricted to analyzing human DNA only. Although it can be adjusted to estimate the depth of any other genome, this requires the availability of a large number of high-quality population samples for filtering.

## Supplementary Material

vbad084_Supplementary_Data

## Data Availability

The data underlying this article are available in the following repositories: RefSeq at https://www.ncbi.nlm.nih.gov/refseq/ with identifiers GCF_009914755.1, GCF_000001635.27 and GCF_000800765.1; GenBank at https://www.ncbi.nlm.nih.gov/genbank/ with identifier CM002798.1; European Nucleotide Archive at https://www.ebi.ac.uk/ena/browser/home with identifier PRJEB54899; 1000 Genomes Project at https://www.internationalgenome.org/.
