# Supplementary material for: DOCEST—fast and accurate estimator of human NGS sequencing depth and error rate
Source: Bioinform Adv. 2023 Jul 18;3(1):vbad084. doi: 10.1093/bioadv/vbad084 (PMC10460481; doi:10.1093/bioadv/vbad084)
Supplement: vbad084_Supplementary_Data [file vbad084_supplementary_data.zip › S1_Explanation_of_the_formulas.pdf]

Explanation of the formulas

We use a simple error model with one universal error probability P_e and equal probabilities for each alternative nucleotide. That is, each position in each read has a probability P_e to be an error, and if it is an error, it has a probability $P_e/3$ to be any one of the wrong nucleotides.
Then we can derive the formulas of expected coverages:

$$D_{tag} = (1 - P_e) D_{kmer}$$

$$D_{err} = P_e \frac{D_{kmer}}{3}$$

D_{kmer} - true k-mer depth of the sample - i.e. the number of all k-mers in the sample, divided by the total number of k-mers in the reference genome. It is less than sequencing depth because k-mers are missed near the end of reads and around N-s.
D_{tag} – observed tag k-mer depth – i.e. the number of tag k-mers seen, divided by the total number of tag k-mers in the list
D_{err} – observed error k-mer depth – i.e. the number of error k-mers seen, divided by the total number of error k-mers in the list
P_e – the rate of sequencing errors (false nucleotide, not N). Here we expect sequencing errors to be unbiased - i.e. each nucleotide in the genome has an equal probability (P_e) to give erroneous value in each read it appears in.

The denominator 3 in the error k-mer depth formula comes from the fact that for each error one tag k-mer is missed but only one of 3 alternative error k-mers is seen. Thus even if 100% of k-mers would contain an error, only ⅓ of the total error k-mers would be seen.

Now we derive the formula for Pe

$$\frac{D_{err}}{D_{tag}} = \frac{P_e \frac{D_{kmer}}{3}}{(1-P_e)D_{kmer}} = \frac{\frac{P_e}{3}}{(1-P_e)}$$

$$\frac{D_{err}}{D_{tag}} (1 - P_e) = \frac{P_e}{3}$$

$$\frac{D_{err}}{D_{tag}} = \frac{P_e}{3} + \frac{P_e D_{err}}{D_{tag}}$$

$$P_e = \frac{\frac{D_{err}}{D_{tag}}}{(\frac{1}{3}+\frac{D_{err}}{D_{tag}})} = \frac{D_{err}}{(\frac{D_{tag}}{3}+D_{err})}$$
